# Early Prediction of Biliary Atresia Using Combi-Elastography in Infants ≤ 60 Days of Age

**DOI:** 10.3390/diagnostics16040571

**Published:** 2026-02-13

**Authors:** Fenglin Xu, Chenpeng Zheng, Caihui Hu, Mingzhu Yu, Xiang Li, Yang Gao, Yi Tang, Jingyu Chen

**Affiliations:** 1 Department of Ultrasound Children’s Hospital of Chongqing Medical University, National Clinical Research Center for Children and Adolescents’ Health and Diseases, Ministry of Education Key Laboratory of Child Development and Disorders, Chongqing Municipal Health Commission Key Laboratory of Children’s Vital Organ Development and Diseases, Chongqing 400014, China; xufenglin1025@163.com (F.X.); zheng_chenpeng@163.com (C.Z.); pangfei9264@163.com (C.H.); 13898122383@163.com (M.Y.); gaoyang577@163.com (Y.G.); 2 Department of Clinical Medicine, School of Clinical Medicine, North Sichuan Medical College, Nanchong 637000, China; m15696232368@163.com

**Keywords:** combi-elastography, biliary atresia, differential diagnosis, children

## Abstract

**Background**: To improve the early recognition of biliary atresia (BA) and timely treatment, this study developed a predictive model integrating combi-elastography, a novel form of elastography, to distinguish biliary atresia from other cholestatic liver diseases (non-BA) in infants. **Method**: A total of 69 children aged < 60 days with cholestatic hepatitis were retrospectively enrolled. All patients underwent conventional ultrasonography, combi-elastography, and laboratory testing. The variables were selected using logistic regression to construct a nomogram model, and the performance of the model was evaluated. **Results**: Multifactorial logistic regression analysis indicated that GGT (*p* = 0.015), the gallbladder morphology (*p* = 0.017), and the fibrosis index of the combi-elastography (*p* = 0.017) could be used as independent predictors to differentiate BA from other causes of cholestasis. A nomogram model constructed with these three indexes showed better performance, with an area under the operating characteristic curve (AUC) of 0.887 (0.823, 0.952) (*p* < 0.001), sensitivity of 86.4%, and specificity of 76.0%. Using 1000 Bootstrap resamples for internal validation of the model, the predictive effect of the nomogram model to identify biliary atresia from other cholestatic liver diseases was in good agreement with the actual situation. Decision-curve analysis showed that the use of the nomogram model to predict biliary atresia gained more clinical value at a risk threshold of 0.10–0.80. **Conclusion**: The nomogram constructed integrating combi-elastography and liver function indices shows promising value for predicting the risk for developing biliary atresia.

## 1. Introduction

Biliary atresia (BA) is characterized by the progressive inflammation and fibrosis of the intrahepatic and extrahepatic bile ducts [1]. Without timely and rational treatment, most children die at about 1 year of age due to liver cirrhosis, portal hypertension, and hepatic failure. It is widely accepted that the timely diagnosis of BA and early surgery (<60 d for Kasai surgery) can significantly improve the prognosis of patients [2]. Therefore, the greatest challenge lies in screening and diagnosing BA as early as possible. However, other cholestatic liver diseases, such as choledochal cysts, Alagille syndrome, and viral infections, exhibit similar clinical manifestations, laboratory findings, and ultrasonographic features with BA, thereby posing substantial difficulties for early differential diagnosis [3,4].

Differentiation of BA from other cholestatic liver diseases can be achieved through clinical manifestations and laboratory tests, such as the presence of clay-like stool and serum gamma-glutamyl transferase (GGT) values. However, although these tests are convenient and easy to perform, they exhibit limited specificity, and individual indicators are readily influenced by various confounding factors [5,6]. Conventional ultrasonography—typically assessing features such as the triangular cord sign and gallbladder morphology—also shows restricted sensitivity [6]. Magnetic resonance cholangiopancreatography (MRCP) and radionuclide hepatobiliary imaging may further assist in distinguishing BA from other cholestatic conditions, yet their diagnostic value is constrained by limitations such as poor intestine visualization or absent tracer excretion [7,8]. Furthermore, endoscopic retrograde cholangiopancreatography (ERCP) and percutaneous transhepatic cholangiography (PTC) can provide a clear visualization of the biliary system and pancreatic ducts, allowing the identification of the location and extent of biliary lesions, but these procedures are invasive with certain risks [9]. Hence, diagnosing BA at an early stage of the disease in a non-invasive, accurate and sensitive way remains an important clinical goal [2].

Currently, an increasing number of studies have utilized ultrasound elastography for the differential diagnosis of BA and other cholestatic liver diseases. However, assessment of liver fibrosis using transient elastography (TE) is easily susceptible to influences from inflammation, cholestasis, and ascites [10]. Nevertheless, the operation of real-time elastography (RTE) is relatively complex, requiring high-quality control and excessive time consumption [11]. Two-dimensional shear wave elastography (2D-SWE) has also been reported to be affected by factors such as obesity, inflammation, and steatosis, with diagnostic performance potentially reduced in the presence of severe inflammation [12]. Moreover, the diagnostic performance of elastography in differentiating between BA and non-BA varies widely among various ultrasound machines from different companies [13].

Combi-elastography, as an emerging technology, integrates both strain and shear wave elastography within a single probe, enabling synchronous real-time assessment on the same imaging plane [14]. In addition to the viscosity (VS) and elasticity (E) values, a variety of quantitative indexes such as liver-fibrosis-related F index (FI), viscosity-related inflammation A index (AI), and attenuation coefficient (ATT) against hepatic steatosis are also obtained, thereby improving accuracy and reproducibility of measurements.

In this study, we proposed using the general information of patients, laboratory examination indexes, and combi-elastography indexes to construct a multifactorial nomogram model that can recognize BA at an early stage, thereby assisting physicians in implementing timely and appropriate treatment.

## 2. Method and Materials

### 2.1. Study Population

We included children with cholestatic liver disease who were examined in the ultrasound department of our hospital from September 2021 to September 2024. Clinical manifestations included worsening jaundice, light-colored stools, fever, etc. Both conventional liver ultrasound and combi-elastography were performed. Laboratory tests, including liver function and routine blood analyses, were obtained within three days after completion of abdominal ultrasonography. This study was approved by the Institutional Review Board of the Hospital (ethical approval number 2024-216; approval date: 15 June 2024), and all examinations and clinical information were conducted after obtaining consent from the parents.

Inclusion criteria: (1) persistent jaundice or persistent aggravation of jaundice; (2) light or white clay-colored stools; (3) elevated serum total bilirubin (TBIL) levels and significantly elevated direct bilirubin (DBIL) levels (TBIL > 28.6 μmol/L, DBIL > 10 μmol/L); (4) first-time consultation without other treatment; (5) children aged < 60 days.

Exclusion criteria: (1) incomplete data; (2) failure of elastography; (3) combination of other serious systemic diseases such as cardiac failure, renal failure, mental illness, etc.; (4) post-liver transplantation; (5) refusal of informed consent by parents or legal guardians.

Finally, a total of 69 children with cholestatic liver disease who met the inclusion and exclusion criteria were enrolled and categorized into the BA group and other cholestatic liver disease group (non-BA group). The diagnosis of the BA group was confirmed through cholangiography and pathological examination of liver biopsy, whereas the diagnosis of the non-BA group was confirmed through cholangiography and pathological examination of liver biopsy that did not support the diagnosis of BA or recovery of cholestasis after conservative treatment.

### 2.2. Laboratory Indexes

Liver function indexes included TBIL, alanine aminotransferase, glutamate transferase, alkaline phosphatase, GGT, and albumin. Blood routine indexes included platelet count test (PLT) and C-reactive protein (CRP) (>8 mg/L was considered inflammation).

### 2.3. Conventional Ultrasound

Ultrasound examinations were performed by an experienced and skilled physician using an ultrasound device (Arietta 850, FUJIFILM Healthcare, Tokyo, Japan) equipped with a convex probe (1–6 MHz, 50 mm radius scan width, 70 field view scan angle, FUJIFILM Healthcare, Tokyo, Japan) operating at a frequency of 2–5 MHz and a high-frequency line array probe. A pacifier was used to keep infants calm during the examination. Sedation was used less frequently and only in a few cases where soothing failed, typically when other procedures (e.g., echocardiography) requiring sedation were being performed during the same session. When necessary, patients were positioned in the supine or left lateral decubitus position to ensure adequate exposure of the right upper abdomen.

Routine ultrasound assessment was performed by two experienced ultrasound doctors who independently assessed the two-dimensional images and recorded measurements. If the assessments were concordant, the results were recorded. Otherwise, reassessment by a third doctor was required. Conventional ultrasound assessment parameters included the size of the liver. Liver enlargement was assessed by measuring the diameter of the right hepatic lobe and the distance of the liver below the right costal margin using a high-frequency ultrasound probe, in combination with the child’s age. If the liver was enlarged, the result was recorded as 1; if not, it was recorded as 0. The gallbladder morphology was defined as abnormal (recorded as “1”) if any of the following features were present: non-visualization, a small lumen, rigid and uneven wall thickness, or a lack of significant contractility between fasting and postprandial states. Conversely, it was defined as normal (recoded as “0”) if the gallbladder exhibited a regular shape with smooth walls and demonstrated noticeable contraction after feeding.

### 2.4. Combi-Elastography Evaluation

**Figure 1:**
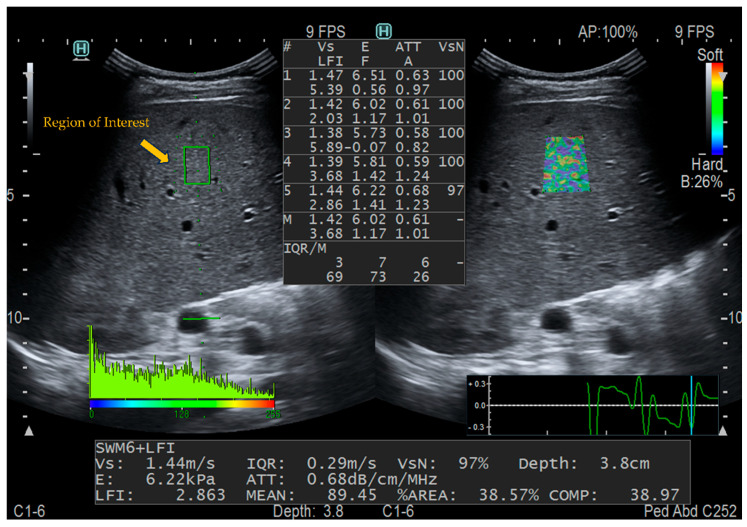
The process of measuring CE in children with cholestatic liver disease. The yellow arrow indicates the region-of-interest (ROI) for elasticity measurement. (Left) The green ROI indicates the sampling area for shear wave elastography measurement (green solid line) and strain elastography measurement (green dashed line). (Right) The color-coded map indicates the ROI of strain elastography. (Center) The table shows the measured parameters integrating both elastography modalities, which are automatically calculated by the built-in ultrasound system, including Vs (m/s), E (kPa), ATT (dB/cm/MHz), LFI, AI (A) and FI (F). VsN is also used as a quality control index for the measurement.

Combi-elastography (CE) examinations were performed on an ultrasound device (Arietta 850, FUJIFILM Healthcare, Tokyo, Japan) with a convex probe (1–6 MHz, 50 mm radius scan width, 70 field view scan angle, FUJIFILM Healthcare, Tokyo, Japan) at a frequency of 2–5 MHz. The CE mode was selected, and the probe was placed vertically on the skin in the right intercostal space. Segment V or VIII sections of the liver were then selected, and a square sampling frame measuring 2.5 × 2.5 cm was positioned within the liver parenchyma at least 1 cm below the liver capsule, while avoiding large hepatic vessels, the gallbladder, and rib shadowing. Measured parameters included E, ATT (to evaluate the extent of steatosis), FI (to denote fibrosis) and AI (to denote inflammation). To ensure the reliability of the results, the percentage of net effective shear wave velocity (VsN) was used as a quality control indicator, i.e., percentage validity. Five measurements of VsN ≥ 50% were taken in each patient [15] and the average of the five measurements was recorded (Figure 1).

### 2.5. Statistical Analyses

SPSS 25.0 statistical analysis software (Armonk, NY, USA) and RStudio (version 2024.04.1, Posit Software, PBC, Boston, MA, USA) were used, and *p* < 0.05 difference was considered statistically significant. The normality of data distribution was assessed using the Shapiro–Wilk test. Normally distributed data was expressed as mean ± standard deviation ($\bar{x\;}$± s) and compared between the two groups by *t*-test, while non-normally distributed data was expressed as median [P25, P75] and compared between the two groups by Mann–Whitney U-test, and counting data was expressed as number of cases (*n*) and percentage (%), and compared between the two groups by chi-square test.

The factors affecting the differential diagnosis of BA and non-BA were determined by LASSO regression and multifactorial logistic analysis. The primary outcome of this study was defined as the discrimination ability of the model between BA and other cholestatic liver diseases. We assessed this outcome using Receiver Operating Characteristic (ROC) curves and the Area Under the Curve (AUC). The secondary outcomes included model calibration, clinical net benefit, and internal validation stability, which were evaluated using calibration curves, Decision Curve Analysis (DCA), and 3-fold cross-validation, respectively.

## 3. Results

### 3.1. General Characteristics

**Figure 2:**
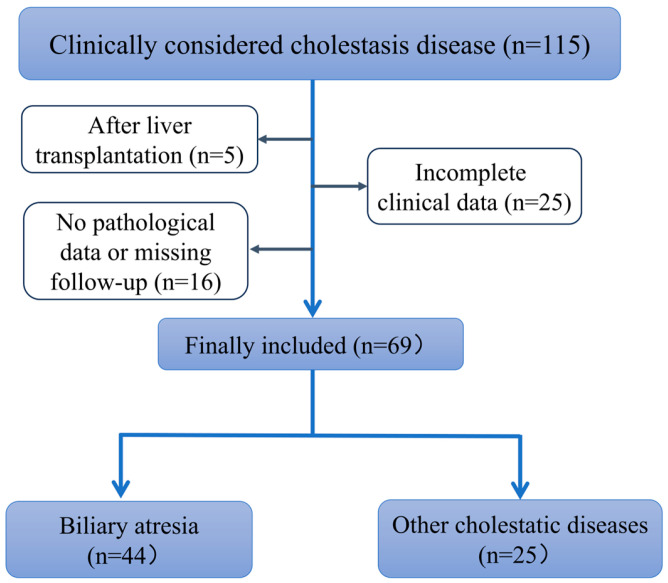
Flow Diagram of Patient Selection and Group Classification.

This study included 69 children with cholestatic hepatitis, 34 males and 35 females, with a mean age of 51.1 (29.2, 62.1) days (Figure 2). According to the basis of grouping, 44 cases were finally included in the BA group and 25 in the group of other cholestatic liver diseases, which included citrin deficiency (*n* = 3), choledochal cysts (*n* = 3), and unexplained jaundice that subsides with conservative treatment (*n* = 19). There were no significant differences between the two groups in terms of age, sex ratio, and several blood test indicators, including aspartate transaminase (AST), alanine aminotransferase (ALT), and alkaline phosphatase (ALP). However, total bilirubin (TBIL) and gamma-glutamyl transpeptidase (GGT) levels were significantly higher in the BA group than in the non-BA group, with statistically significant differences. Regarding ultrasonographic parameters, there was no significant difference in liver enlargement between the two groups. In contrast, the CE indices (E, FI, and AI) were significantly higher in the BA group compared with the non-BA group. Additionally, abnormal gallbladder morphology was more common in the BA group (65.90%) than in the other cholestatic groups. The demographic, laboratory, and ultrasonographic data of the two groups are summarized in Table 1.

### 3.2. Nomogram Construction

**Figure 3:**
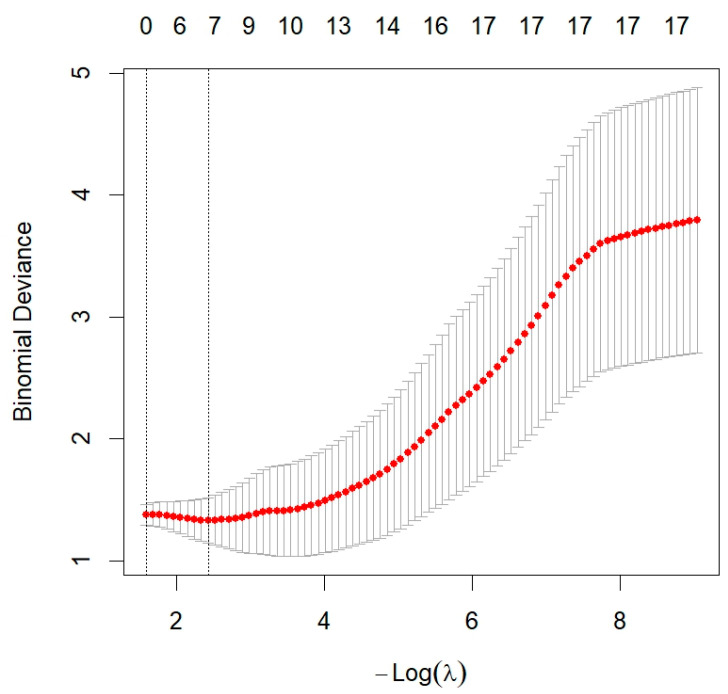
LASSO Cross-Validation Plot for Selecting the Optimal λ Value. The red dots represent the mean cross-validated error for each λ, and the vertical bars indicate the standard deviation. The left dotted line denotes λ_min_, while the right dotted line denotes λ_1se_.

**Figure 4:**
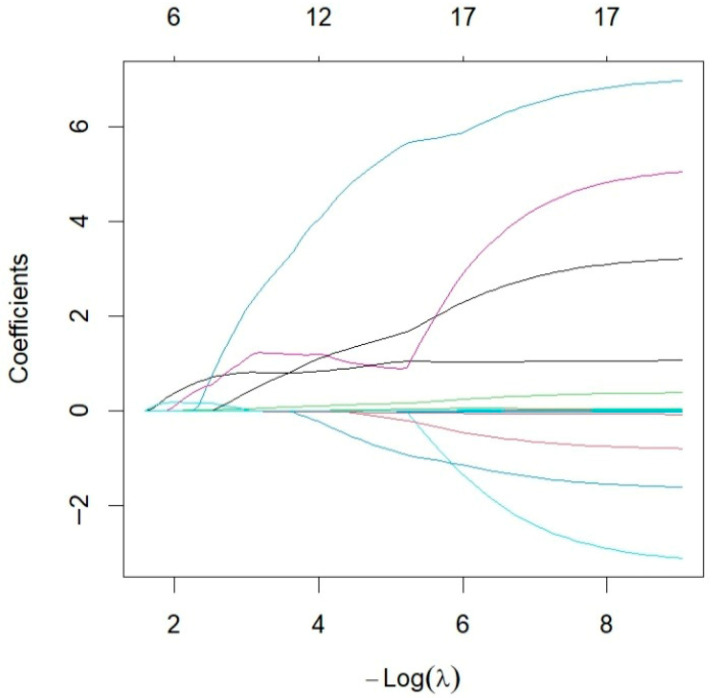
LASSO Coefficient Profiles Across Log(λ) Values. Each colored line represents the coefficient trajectory of an individual variable as λ changes.

The data were filtered using LASSO regression (10-fold cross-validation, optimal λ = 0.087), resulting in seven candidate variables: Gallbladder morphology, E, ATT, FI, AI, TBIL, GGT (Figure 3 and Figure 4). Spearman correlation analysis revealed strong positive correlations among E, FI, and AI (all r > 0.85, *p* < 0.001). Collinearity diagnostics further indicated a high variance inflation factor (VIF) for AI (9.861), with E exhibiting the second-highest VIF (6.762). In addition, both E and FI are indicators used to assess hepatic fibrosis. Therefore, AI and E were excluded to optimize model stability (Supplementary Tables S1 and S2).

**Figure 5:**
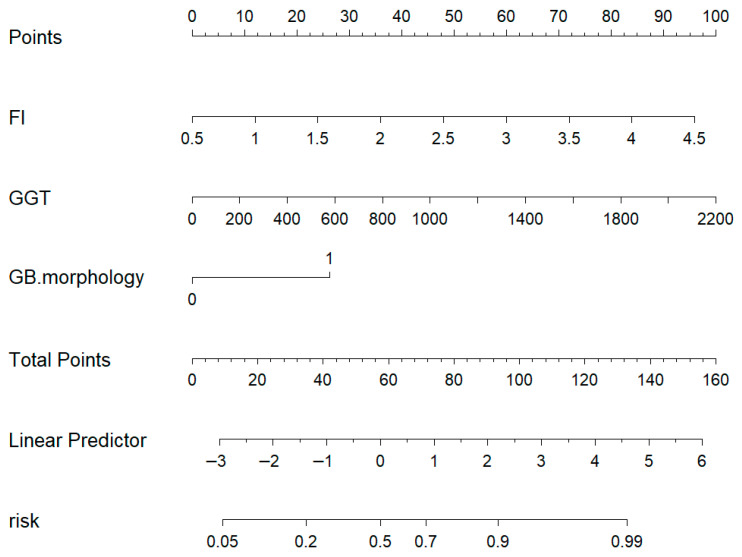
Nomogram model of biliary atresia based on GGT, FI, and GB morphology.

Although TBIL exhibited borderline statistical significance (*p* = 0.05), it owned a relatively low odds ratio (OR = 1.013) and had less clinical specificity than GGT for biliary atresia. Ultimately, the FI values, GGT, and gallbladder morphology were the independent predictors used to distinguish BA from other cholestatic liver diseases; the odds ratio (OR) for FI was 3.542 (1.257, 9.984), *p* = 0.017, the OR for GGT was 1.003 (1.001, 1.026), *p* = 0.015, and the OR for gallbladder morphology was 5.878 (1.376, 25.114), *p* = 0.017 (Table 2). To further visualize the data for clinical use, a nomogram model was constructed based on the GGT, FI, and gallbladder morphology values (Figure 5).

In a pediatric patient suspected of biliary atresia, the combination of laboratory testing and ultrasound elastography showed an FI value of 1.75 (30 points), GGT of approximately 700 U/L (32 points), and abnormal gallbladder morphology (25 points), with a total score of 87, indicating an estimated biliary atresia risk of about 85%.

### 3.3. Diagnostic Performance of Laboratory and Ultrasound Indexes

**Figure 6:**
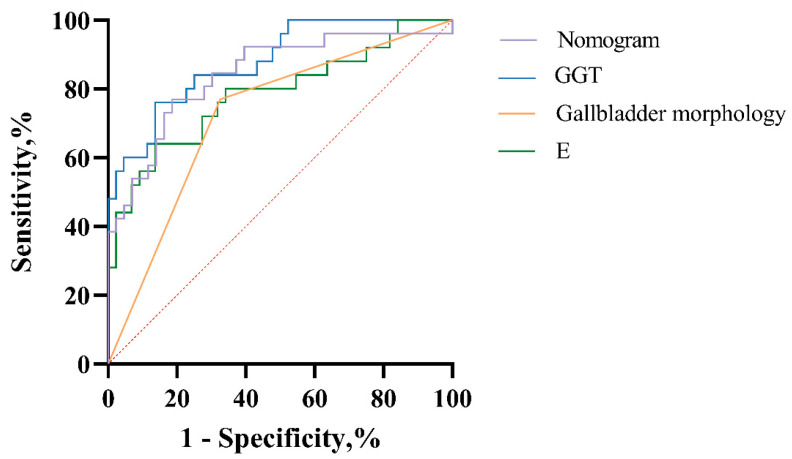
ROC curve of CE indexes, serum biomarkers, and models in diagnosis of biliary atresia. The red dotted line represents the reference line (AUC = 0.5).

ROC curve analysis was performed to evaluate the independent predictors and the combined diagnostic model for distinguishing BA from other cholestatic liver diseases. Diagnostic performance was assessed using the area AUC. The AUC areas of E for distinguishing BA from other cholestatic liver diseases were 0.785 (0.664, 0.905). The AUC areas of GGT were 0.819 (0.735, 0.902). Gallbladder morphology demonstrated an AUC of 0.851 (0.770, 0.933). Notably, the nomogram model achieved the highest diagnostic performance, with an AUC of 0.887 (95% CI: 0.823–0.952), along with superior sensitivity and specificity compared with the individual predictors. (Table 3, Figure 6).

### 3.4. Evaluation of Nomogram Performance

**Figure 7:**
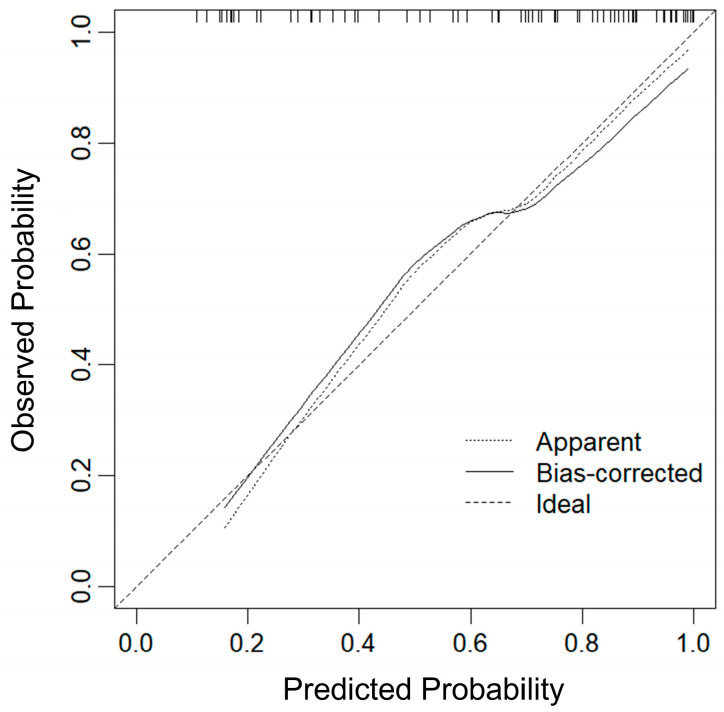
Calibration curve of nomogram model.

As seen in Figure 7, internal validation of the nomogram model using 1000 bootstrap resamples demonstrated good calibration. The calibration curve closely approximated the 45° reference line, indicating strong agreement between the predicted probabilities and the actual outcomes. This suggests that the nomogram exhibits excellent predictive performance in distinguishing BA from other cholestatic liver diseases.

**Figure 8:**
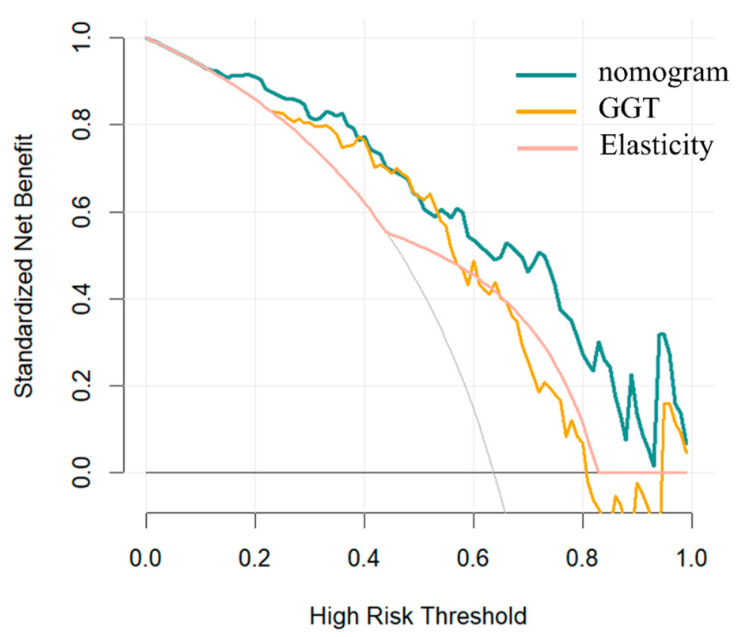
Decision-curve analysis of nomogram model. The grey line represents the strategy of “intervention for all”, and the horizontal line represents “intervention for none”.

Decision curve analysis (DCA) (Figure 8) demonstrated that the nomogram model provided greater clinical value and yielded a higher net benefit in distinguishing BA from other cholestatic liver diseases compared with GGT and elasticity. Furthermore, to evaluate the robustness of the nomogram model, a three-fold cross-validation was performed. The AUC values across the three folds were 0.86, 0.84, and 0.84, and the corresponding accuracies were 0.83, 0.78, and 0.80, respectively. The average AUC and accuracy were 0.85 and 0.80, indicating that the model maintained stable predictive performance.

## 4. Discussion

In this study, data from 69 children with cholestatic hepatitis were analyzed, and a nomogram model was constructed based on the combi-elastography indexes FI, GGT, and gallbladder morphology. The nomogram demonstrated excellent diagnostic performance, achieving an AUC of 0.887 (95% CI: 0.823–0.952). Multiple validation approaches further confirmed the robustness and stability of the model. Moreover, compared with combi-elastography using a single E-value, the nomogram exhibited superior discriminative ability, highlighting the advantage of integrating multiparametric indicators rather than relying on a single stiffness measurement. We further evaluated the calibration and clinical utility of the nomogram. The calibration curve indicated good agreement between predicted and observed probabilities. Decision-curve analysis demonstrated that the nomogram provided the greatest net clinical benefit across a wide range of threshold probabilities (0.10–0.80), covering the clinically relevant range for guiding further diagnostic interventions. These findings suggest that, in routine practice, applying the nomogram may facilitate the identification of high-risk infants who would benefit from timely invasive procedures, while simultaneously reducing unnecessary investigations in low-risk individuals, thereby optimizing the diagnostic pathway for biliary atresia.

Unlike previous studies, this study employed a novel elastography device whose value in liver assessment has been reported in prior research. Zhao Y et al. applied CE to predict early recurrence after radical hepatectomy in adults and identified the fibrosis index (FI) as an independent predictor [14]. Another study evaluating fibrosis staging and inflammatory activity in adults with chronic liver disease demonstrated that CE exhibited significantly higher diagnostic performance than either shear wave elastography or strain elastography alone [16]. In pediatric populations, CE has proven effective for assessing liver fibrosis and monitoring postoperative recovery after liver transplantation [17,18]. Collectively, these studies suggest that CE provides a superior assessment of liver conditions. However, limited studies have applied this technology to BA and other cholestatic liver diseases. Therefore, this study constructed a nomogram model based on the CE indexes and laboratory indexes which further supplemented the existing literature and provided a stronger theoretical basis for related studies.

The laboratory index GGT demonstrated relatively good diagnostic efficacy in distinguishing BA from other cholestatic liver diseases. A meta-analysis reported that the sensitivity and specificity of GGT for diagnosing BA were 81.5% and 72.1%, respectively [19]. Moreover, elevated GGT significantly associated with advanced hepatic fibrosis, further underscores its utility as a reliable biomarker in pediatric liver evaluation [20]. Similarly, abnormal gallbladder morphology is considered an important ultrasound feature for the diagnosis of biliary atresia, with reported sensitivity and specificity of 85% and 92%, respectively [21]. However, accurate identification of abnormal gallbladder morphology remains somewhat subjective and may be more challenging for less experienced operators [22,23]. It is worth noting that sex and AST exhibited borderline significance in univariate analysis but were excluded through LASSO screening to prioritize model parsimony. While the observed female predominance aligns with known BA epidemiological patterns [24], its borderline statistical significance in this study likely due to the limited sample size. GGT was retained over AST owing to its superior specificity for biliary obstruction rather than general hepatocellular injury. Therefore, in the present study, GGT and gallbladder morphology were incorporated with FI value, to enhance the diagnostic capability for distinguishing BA from other cholestatic liver diseases, and the nomogram we constructed showed superior performance over other single indicators owing to the integrated contribution of multiple predictors.

Although several existing relevant studies have used predictive models for differential diagnosis of BA and other cholestatic liver diseases, this study further improved on this basis. Chen Y et al. [25] created a three-color hierarchical analysis for the risk assessment of BA; however, the nomogram model can provide individualized, specific risk probabilities for BA, enabling more precise clinical decision making. Furthermore, unlike earlier models that relied solely on serum biomarkers [26] or shear-wave elastography (SWE) [27] combined with conventional ultrasound, our nomogram integrates both ultrasonographic indicators—including elastography parameters—and key biochemical markers such as GGT, thereby offering a more comprehensive and informative diagnostic assessment. Additionally, Yan H et al. [28] constructed a nomogram based on SWE, notably, the combi-elastography technique we used integrates shear-wave elastography and strain elastography. The Fibrosis Index (FI) is a combined metric derived from a multiple linear regression model integrating both real-time elastography and shear-wave elastography [16], further enhancing the diagnostic capability of single shear-wave elastography in the presence of inflammation and jaundice.

This study has several strengths. First, we employed the innovative CE technique to assess liver fibrosis. CE enables simultaneous real-time elastography and shear-wave elastography, allowing the two modalities to complement each other’s limitations. This technique has rarely been applied in the diagnostic evaluation of BA; therefore, our findings may provide a stronger theoretical basis for future research. Second, we developed a nomogram model intended to distinguish BA from the collective pool of other cholestatic disease incorporating both combi-elastography and liver function indices to estimate the risk of BA in children. This model demonstrated superior diagnostic performance and accuracy, offering more precise and comprehensive guidance for clinical decision making and facilitating timely and appropriate management for affected patients.

However, this study has several limitations. First, the sample size was relatively small and derived from a single center, although we intentionally restricted the age range to infants younger than 60 days to maximize the model’s early diagnostic performance, which inevitably reduced the number of eligible participants. Consequently, the nomogram model should currently be interpreted as an exploratory or hypothesis-generating tool rather than a model ready for immediate clinical implementation. Furthermore, the high prevalence of BA observed in a tertiary referral setting may influence model calibration in low-prevalence populations, potentially leading to risk overestimation. Therefore, future large-scale, multi-center external validation and recalibration are essential to ensure generalizability and to evaluate the model’s performance within specific clinically relevant subgroups. Second, additional biomarkers could be incorporated to improve diagnostic performance. For example, matrix metalloproteinase-7 (MMP-7), which has been reported to have high specificity for BA, may further enhance the predictive accuracy of the nomogram in future work.

## 5. Conclusions

Our results indicated that the nomogram model based on CE and liver function indices has good diagnostic efficacy and can be applied to the preoperative differential diagnosis of BA and non-BA. In conclusion, CE offers valuable support for clinicians in distinguishing BA from other causes of cholestasis, enabling timely and appropriate clinical management and ultimately helping pediatric patients achieve the greatest possible clinical benefit.

## Figures and Tables

**Table 1:** Clinical information of biliary atresia and other cholestatic liver disease groups.

	All Children	BA	Non-BA	*p*
Age, days	51.1 (29.2, 62.1)	47.9 (20.2)	52.9 (20.6)	0.323
Sex, *n* (%)				0.065
Male	34 (49.30%)	18 (40.90%)	16 (64.00%)	
Female	35 (50.70%)	26 (59.10%)	19 (36.00%)	
AST, U/L	200.0 (128.00, 277.00)	225.50 (145.75, 298.00)	172.00 (102.00, 252.00)	0.060
ALT, U/L	131.0 (84.00, 234.00)	128.00 (87.25, 248.25)	134.00 (71.50, 222.50)	0.644
TBIL, μmol/L	157.52 (64.10)	173.01 (57.16)	130.26 (67.64)	0.007
ALP, U/L	534.00 (430.00, 670.00)	543.61 (152.92)	567.32 (218.91)	0.599
GGT, U/L	278.00 (131.50, 489.50)	386.00 (246.25, 614.25)	128.00 (62.50, 214.00)	<0.001
TP, g/L	57.60 (7.01)	58.09 (5.98)	56.73 (8.59)	0.442
ALB, g/L	40.49 (5.37)	40.76 (5.02)	40.02 (6.00)	0.583
PLT, 109/L	418.05 (144.48)	422.98 (142.25)	421.00 (352.00, 501.00)	0.100
CRP, mg/L	20 (29.00%)	13 (29.5%)	7 (28.00%)	0.892
Liver enlargement, *n* (%)	34 (49.30%)	22 (50.00%)	12 (48.00%)	0.873
E, kPa	12.43 (9.04, 161.17)	13.83 (11.00, 17.63)	8.89 (6.24, 12.39)	<0.001
ATT, dB/cm/MHz	0.61 (0.53, 0.66)	0.609 (0.09)	0.6 (0.10)	0.062
FI	2.35 (0.77)	2.59 (0.72)	1.92 (0.70)	<0.001
AI	1.47 (0.32)	1.569 (0.29)	1.30 (0.30)	<0.001
Gallbladder morphology	35 (50.70%)	29 (65.90%)	6 (24.00%)	0.001

AST: Aspartate Transaminase, ALT: Alanine Aminotransferase, TBIL: Total Bilirubin, ALP: Alkaline Phosphatase, GGT: Gamma-Glutamyl Transferase, TP: Total Protein, ALB: Albumin, PLT: platelet count test, CRP: C-reactive protein, E: Elasticity, ATT: Attenuation coefficient, FI: F index, AI: A index.

**Table 2:** Multivariate logistic regression of factors in diagnosis of biliary atresia.

Variable	β	SE	OR (95% CI)	*p*
FI	1.265	0.529	3.542 (1.257, 9.984)	0.017
GGT	0.003	0.001	1.003 (1.001, 1.026)	0.015
TBIL	0.013	0.006	1.013 (1.000, 1.026)	0.050
Gallbladder morphology	1.771	0.741	5.878 (1.376, 25.114)	0.017
ATT	5.584	3.297	266.017 (0.415, 170, 404.681)	0.090

FI: F index, GGT: Gamma-Glutamyl Transferase, TBIL: Total Bilirubin, ATT: Attenuation coefficient.

**Table 3:** Diagnostic performance of CE indexes, serum biomarkers, and models in diagnosis of biliary atresia.

Variables	Cutoff-Value	AUC (95% CI)	Sensitivity, %	Specificity, %	*p*
E	10.055	0.785 (0.664, 0.905)	0.864	0.640	<0.001
GGT	232	0.819 (0.735, 0.902)	0.818	0.80	<0.001
Gallbladder morphology	—	0.851 (0.770, 0.933)	0.659	0.76	<0.001
Nomogram	—	0.887 (0.823, 0.952)	0.864	0.76	<0.001

## Data Availability

Data are available from the corresponding author Jingyu Chen (Email: cjy419103@163.com).

## Supplementary Materials

The following supporting information can be downloaded at: https://www.mdpi.com/article/10.3390/diagnostics16040571/s1, Table S1: Correlation Analysis and Collinearity Diagnostics of Combi-Elastography Parameters; Table S2: Collinearity Diagnostics of Indicators After Excluding E and AI.
