# Supplementary material for: Early Prediction of Biliary Atresia Using Combi-Elastography in Infants ≤ 60 Days of Age
Source: Diagnostics (Basel). 2026 Feb 13;16(4):571. doi: 10.3390/diagnostics16040571 (PMC12939678; doi:10.3390/diagnostics16040571)
Supplement: Supplementary file 1 [file diagnostics-16-00571-s001.zip › diagnostics-4118973-supplementary.pdf]

Table S1. Correlation Analysis and Collinearity Diagnostics of Combi-Elastography Parameters

Variable	r	*P*		Variable	Variance Inflation Factor
E vs FI	0.854	< 0.001		AI	9.861
E vs AI	0.898	< 0.001		E	6.762
FI vs AI	0.902	< 0.001		FI	6.033

E: Elasticity, ATT: Attenuation index, FI: F index, AI: A index

Table S2. Collinearity Diagnostics of Indicators After Excluding E and AI.

Variable	B	Variance Inflation Factor	*P*
FI	0.169	1.108	0.011
TBIL	0.001	1.076	0.092
GGT	0.000	1.063	0.019
Gallbladder morphology	0.268	1.106	0.008
ATT	0.973	1.041	0.035

GGT: Gamma-Glutamyl Transferase, TBIL: Total Bilirubin, E: Elasticity, ATT: Attenuation index, FI: F index, AI: A index
